# Chitosan-Based Thermo-Sensitive Hydrogel Loading Oyster Peptides for Hemostasis Application

**DOI:** 10.3390/ma13215038

**Published:** 2020-11-09

**Authors:** Dongying Zhang, Zhang Hu, Lingyu Zhang, Sitong Lu, Fengyan Liang, Sidong Li

**Affiliations:** 1 Faculty of Chemistry and Environmental Science, Guangdong Ocean University, Zhanjiang 524088, China; 17875128648@163.com (D.Z.); zhangly175@163.com (L.Z.); smelilst@163.com (S.L.); yaner204126@163.com (F.L.); sidongligdou@163.com (S.L.); 2 Southern Marine Science and Engineering Guangdong Laboratory (Zhanjiang), Zhanjiang 524088, China

**Keywords:** chitosan, oyster peptides, hydrogel, hemostasis

## Abstract

Uncontrolled massive hemorrhage is one of the principal causes of death in trauma emergencies. By using catechol-modified chitosan (CS-C) as the matrix material and β glycerol phosphate (β-GP) as a thermo-sensitive agent, chitosan-based thermo-sensitive hydrogel loading oyster peptides (CS-C/OP/β-GP) were prepared at physiological temperature. The hemostatic performance of CS-C/OP/β-GP hydrogel was tested in vivo and in vitro, and its biological safety was evaluated. The results showed that the in vitro coagulation time and blood coagulation index of CS-C/OP/β-GP hydrogel were better than those of a commercial gelatin sponge. Notably, compared with the gelatin sponge, CS-C/OP/β-GP hydrogel showed that the platelet adhesion and erythrocyte adsorption rates were 38.98% and 95.87% higher, respectively. Additionally, the hemostasis time in mouse liver injury was shortened by 19.5%, and the mass of blood loss in the mouse tail amputation model was reduced by 18.9%. The safety evaluation results demonstrated that CS-C/OP/β-GP had no cytotoxicity to L929 cells, and the hemolysis rates were less than 5% within 1 mg/mL, suggesting good biocompatibility. In conclusion, our results indicate that CS-C/OP/β-GP is expected to be a promising dressing in the field of medical hemostasis.

## 1. Introduction

Rapid hemostasis in emergencies has great clinical significance for patients with massive and potentially fatal hemorrhage caused by war, operations, accidents, or hematologic disease [1]. Hemostasis is an important step in emergency medical treatment [2]. At present, many kinds of hemostatic materials are available on the market. While they have obvious hemostatic effects, their shortcomings are considerable; for example, collagen dressings have poor tissue adhesion [3]; porous zeolite releases heat after absorbing water in the blood, which may lead to wound inflammation [4]; and carboxymethyl gauze cannot be degraded on the wound surface, which can create scars following removal [5].

As a natural polycation polysaccharide prepared by chitin deacetylation, chitosan is characterized by excellent coagulation, bacteriostasis, a film-forming ability, wound healing promotion, and scar formation inhibition. In addition to these features, it is a multifunctional material with good biocompatibility, no immunogenicity, and no dermal irritation [6,7,8]. The primary hemostatic mechanism of chitosan is that it carries a small positive charge, promoting red blood cell aggregation and platelet adhesion [9,10]. However, the hemostatic effect of chitosan is not sufficient to treat severe bleeding wounds [11]. Many researchers have studied how to improve its hemostatic potential. The chemical modification of chitosan and its combination with other active ingredients can enhance the hemostatic effect and achieve rapid hemostasis [12,13,14,15]. Cheng et al. [16] prepared a chitosan/gelatin thermo-sensitive hydrogel with β-glycerophosphate as the thermo-sensitive agent. The blend significantly improved the survival rate of encapsulated adipose-derived stem cells, providing a promising method to accelerate the regeneration of ischemic tissue. Zhao et al. [17] successfully prepared a new type of thermo-sensitive chitosan hydrogel loaded with β-cyclodextrin and curcumin with an inner porous structure that enables sustained curcumin release. In a rat wound infection model, the hydrogel exhibited a significant hemostatic effect and wound healing ability.

Biological resources are abundant in oceans, and numerous marine products harbor natural active substances with unique structures and powerful functions. Among these, marine active peptides are an essential part of investigations into aquatic active substances. Some evidence suggests that marine active peptides have antibacterial, hemostatic, and wound healing-promoting functions [18,19,20]. Oyster peptides (OP) from marine organisms are composed of active substances, such as antibacterial peptides, angiotensin I converting enzyme inhibitor peptides, and antioxidant peptides [21,22]. Studies have shown that OP have special biological activities, such as antibacterial, antioxidant, hypoglycemic, anti-aging, and anti-tumor activities [23,24]. OP also display some typical physical and chemical characteristics, such as small molecular weights, easy absorption, and non-toxic and harmless natures [21,22]. Furthermore, due to their abundant nutrition and poor biostability, OP are vulnerable to microorganism attack, leading to the loss of their biological function [25]. On the other hand, OP are water-soluble and cannot be directly used to stop bleeding. To make full use of the benefits of catechol-functionalized chitosan (CS-C)-based hydrogel and OP to obtain an effective hemostatic dressing, in this study, with CS-C as the matrix material, chitosan-based thermo-sensitive hydrogel loading oyster peptides (CS-C/OP/β-GP) were prepared. Furthermore, their hemostatic efficacy and biological safety in vivo and in vitro were investigated.

## 2. Materials and Methods

### 2.1. Materials

Chitosan (DD ≥ 85% and MW 100 kDa) was used to prepare CS-C by our laboratory [26]. Oyster peptides (about 45% total protein content, batch NO. 20181018B) were purchased from Hainan Semnl Biotechnology Co., Ltd. (Hainan, China) and the molecular weight of most peptides (>95%) was less than 1000 Da. The mouse fibroblast cell line L929 was provided by the Kunming Cell Bank of the Chinese Academy of Sciences, Kunming, China and the passage numbers used in the experiment were 3-10. Dulbecco’s Modified Eagle Medium (DMEM), fetal bovine serum (FBS), antibiotics (penicillin and streptomycin), trypsin, and PBS (0.01 M, pH = 7.4) were all purchased from Gibco, Grand Island, NY, USA. Calcein-AM/PI and AnnexinV-FITC/PI kits were commercially obtained from Shanghai Yisheng Biotechnology Co., Ltd. (Shanghai, China). Thiazolyl blue tetrazolium bromide (MTT) and dimethyl sulfoxide were acquired from Shanghai Aladdin Biochemical Technology Co., Ltd. (Shanghai, China). β-GP and 1% Triton-X100 were purchased from SIGMA (Milwaukee, Wisconsin, USA). Lactate dehydrogenase (LDH) kits were purchased from Shanghai Yuanye Biotechnology Co., Ltd. (Shanghai, China). All other chemical agents were of analytical grade.

### 2.2. Preparation and Characterization of Chitosan-Based Thermo-Sensitive Hydrogel

CS-C was dissolved in 100 mL distilled water to obtain a 2% CS-C aqueous solution, into which 0.1 g OP was added and mixed. Under stirring, the 30% β sodium glycerophosphate (β-GP) solution (volume ratio of CS-C to β-GP was 8:2) was added dropwise, and then placed in a 37 ℃ water bath to produce CS-C/OP/β-GP thermo-sensitive hydrogel and CS-C/β-GP thermo-sensitive hydrogel without OP.

The samples were sprayed with gold under vacuum, and then placed under a scanning electron microscope (SEM, S-4800, Tokyo, Japan) with a voltage of 10 kV.

### 2.3. Determination of Water Absorption

The hydrogel mass after freeze-drying was recorded as $m_{1}.$ The hydrogel was soaked in distilled water for 10 min and then taken out, and the free water from the surface of the hydrogel was extracted with filter paper. The hydrogel was weighed again as $m_{2}$. Tests in each group were repeated three times, and the values were averaged. The calculation equation of water absorption (WA) is as follows:

(1) $$\mathrm{WA}=\frac{m_{2}-m_{1}}{m_{1}}\times 100\%,$$

where WA is the water absorption (%), and $m_{1}\;$ and $m_{2}\;$ are hydrogel masses (g) before and after soaking in water, respectively.

### 2.4. In Vitro Hemostasis Evaluation

#### 2.4.1. Blood Coagulation Index (BCI)

New Zealand white rabbits were provided by the Guangdong Medical Laboratory Animal Center, Sanshui base (Certificate No. SCXK 20180002, Foshan, China). New Zealand rabbit blood was collected with evacuated blood tubes (sodium citrate as an anticoagulant to blood ratio of 1:9, *v*/*v*) and kept at 4 °C for use. A 10 mg sample was placed into a beaker in a 37 °C thermostatic water bath, and 100 μL anticoagulated rabbit blood and 20 μL 0.2 mol/L CaCl_2_ solution were then added, followed by 25 mL distilled water after 5 min. The sample was shaken well at 37 °C and the absorbance was measured at 545 nm. The control group did not contain samples, and the average value of three samples in each group was measured in parallel.

(2) $$\mathrm{BCI}=\frac{A_{\mathrm{sample}}}{A_{\mathrm{control}}}\times 100\%,$$

where A_sample_ is the absorbance value of each experimental sample and A_control_ is the absorbance value of the control group without adding the sample.

#### 2.4.2. In Vitro Coagulation Time

Samples (10 mg) were placed into a 2 mL vial and preheated for 5 min with a 37 °C water bath, and 0.5 mL anticoagulated rabbit blood was added. The mixture was heated in the water bath for 5 min, and then 100 μL 0.1 mol/L CaCl_2_ solution was added. The vial was tilted every 10 s to observe whether the mixture had coagulated, and the coagulation time was recorded.

#### 2.4.3. Platelet Adhesion

According to the method of Ong et al. [27], the samples were cut into 0.4 cm × 0.4 cm × 0.2 cm pieces and added to 50 μL platelet-rich plasma, before being incubated at 37 °C for 1 h. Then, the samples were washed twice with PBS to remove non-adherent platelets. The control group did not contain the sample. The samples were placed in 1% Triton-X100 solution at 37 °C for 1 h to cleave adherent platelets, and were then detected according to the LDH kit instructions. In order to observe the adhesion state of platelets, the samples were fixed in 2.5% glutaraldehyde for 30 min, before being dehydrated with 30%, 40%, 50%, 60%, 70%, 80%, 90%, and 100% ethanol for 15 min successively; dried naturally; sprayed with gold for sample preparation; and observed and photographed by SEM.

#### 2.4.4. Erythrocyte Adsorption

Fresh anticoagulated rabbit blood was centrifuged at 2000 rpm for 15 min to separate the red blood cells which were diluted to 10% with PBS. Next, 500 μL of the red blood cells was added into the samples of 0.4 cm × 0.4 cm × 0.2 cm and incubated at 37 °C for 1 h. The samples were washed twice with PBS to remove non-adherent red blood cells, and then placed in 1% Triton-X100 solution at 37 °C for 1 h to split the adherent red blood cells. Absorbance was measured at 540 nm. The control group did not contain the sample, and the blank control group did not contain red blood cells. In order to observe the adsorption state of red blood cells, the samples were fixed in 2.5% glutaraldehyde for 30 min; dehydrated with 30%, 40%, 50%, 60%, 70%, 80%, 90%, and 100% ethanol for 15 min in turn; and then dried naturally. Following this, the samples were sprayed with gold, observed, and photographed by SEM.

(3) $$Erythrocyte\;Adsorption=\frac{A_{\mathrm{sample}}}{A_{\mathrm{control}}}\times 100\%,$$

where A_sample_ and A_control_ are the absorbance values of the sample and control groups without sample, respectively.

### 2.5. Evaluation of Animal Hemostasis

#### 2.5.1. Animal Preparation

Male Kunming mice weighing 30–40 g were purchased from Guangdong Medical Experimental Animal Center (Foshan, China). Before the experiment, they were acclimated in the laboratory for 1 week to adapt to the environment. All the animal experiments were approved by the Animal Care and Use Committee of Guangdong Ocean University, China (SYXK20180147).

#### 2.5.2. Mouse Liver Hemorrhage

According to methods in the references [27,28,29], the mice were anesthetized, and the abdomens were shaved and disinfected with 75% alcohol. After the abdominal cavity was carefully cut, a liver section was taken out to create a wound about 0.5 cm long, and bleeding occurred spontaneously for 5 s. The blood was wiped with gauze, and the sample was gently pressed on the wound. The bleeding status of the wound was observed, and the time was recorded when the wound stopped bleeding. The hemostatic dressing was weighed to calculate the amount of blood, and the average value of six parallel experiments was adopted.

#### 2.5.3. Histological Analysis of the Mouse Liver

Two hours after hemostasis, liver tissue of the bleeding site was collected, and the hemostatic and blood were removed with normal saline. The tissue was fixed with 4% glutaraldehyde, embedded in paraffin, sectioned, stained with hematoxylin and eosin (H&E), and observed under an optical microscope.

#### 2.5.4. Mouse Tail Amputation Hemorrhage

The tails of anesthetized mice were sterilized with 75% alcohol to make the tail hyperemic. Next, 50% of the tail was cut off and allowed to bleed for 15 s. The tail tissue was put into a small centrifuge tube with the sample so that the wound was completely covered by the sample. The time was recorded when the tail stopped bleeding. The blood was collected with standard medical gauze that was weighed to calculate the blood loss. The average value was acquired after six parallel experiments.

### 2.6. Safety Evaluation

#### 2.6.1. Cytotoxicity

The cytotoxicity of hydrogel was assessed using the L929 cell line. L929 cells were cultured in DMEM containing 10% fetal bovine serum (FBS) and antibiotics (streptomycin 100 U/mL and penicillin 100 U/mL) at 37 °C under a humidified atmosphere of 95% air and 5% CO_2_. After cell counting, the L929 cell concentration was adjusted to 5 × 10^4^ /mL. The cells were inoculated in a 96-well culture plate and placed in an incubator at 37 °C with 5% CO_2_ for 24 h. Next, they were treated by 50 μL DMEM with different concentrations of samples, and the group without samples was the control group. They were further incubated for 24 or 48 h. MTT assays were employed to measure the cell viability.

A previously described method [30] was adopted to distinguish the dead and alive states of cells. After being cultured in a 24-well culture plate, L929 cells were treated with the samples, and the culture medium was then aspirated and discarded. The cells were washed twice with PBS, and 500 μL Calcein-AM/PI solution (5 μL Calcein AM and 15 μL PI in 5 mL 1×assay Buffer) was added. The mixture was stained in the dark for 20 min and washed once with PBS after the staining solution was discarded, and 500 μL culture medium was added. Photos were taken under a fluorescent microscope.

#### 2.6.2. Apoptosis Experiment

According to the reference [31], L929 cells were cultured in a 6-well culture plate and digested with trypsin after treatment with the samples, before being centrifuged at 850 rpm for 5 min. The cells were collected and washed twice with pre-cooled PBS, and then centrifuged again. Next, 100 μL 1×Binding Buffer (Yisheng Corporation, Shanghai, China) was used to resuspend the cells, and 5 μL Annexin V-FITC and 10 μL PI Staining Solution (Yisheng Corporation, Shanghai, China) were added. The mixture was kept in the dark at room temperature for 10 min, and 400 μL 1×Binding Buffer was added for measuring by flow cytometry.

#### 2.6.3. Hemolysis Analysis

Sample solution (500 μL) was added into a 1.5 mL centrifuge tube and placed in a 37 °C thermostatic water bath for 30 min. Next, 500 μL 2% red blood cell suspension was added, and the mix was cultivated for 30 min in a 37 °C thermostatic water bath. Distilled water was used as a positive control and PBS as a blank control. The test was repeated three times in each group. The mixture was centrifuged at 2000 rpm and 4 °C for 15 min, and the supernatant was measured at 540 nm to determine its absorbance and calculate the hemolysis rate. To observe morphological changes in red blood cells after treatment, the centrifuged red blood cells were collected and resuspended with PBS, and the morphology was observed and recorded under an optical microscope. The hemolysis rate was calculated as follows:

(4) $$Hemolysis\;rate(\%)=\frac{H-H_{0}}{H_{100}-H_{0}}\times 100\%,$$

where *H*, *H*_0_, and *H*_100_ are the absorbance of the sample group, PBS group, and distilled water group, respectively. 

### 2.7. Statistical Analysis

All the data were given as the mean ± standard deviation (X ± SD). The data were statistically processed with SPSS 17.0 statistical software (International Business Machines Corporation, Armonk, NY, USA) and analyzed using Student’s t-tests. Statistical comparisons were performed by using one-way analysis of variance (ANOVA) to demonstrate differences between groups. Differences were considered significant at * *p* < 0.05 and highly significant at ** *p* < 0.01.

## 3. Results and Discussion

### 3.1. Microstructure of CS-C/OP/β-GP

**Figure 1:**
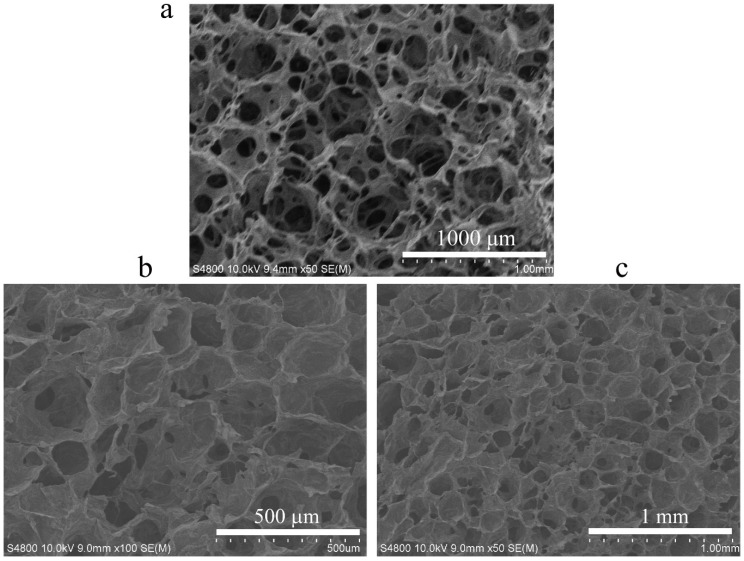
Scanning electron microscope (SEM) images of commercial gelatin sponge (**a**) and chitosan-based thermo-sensitive hydrogel loading oyster peptide (CS-C/OP/β-GP) hydrogels (**b**,**c**).

SEM revealed that the hydrogel CS-C/OP/β-GP had an even porous network structure similar to the commercial gelatin sponge with an average pore size of 100–200 μm (Figure 1). Its structure enables it to absorb water in large quantities and concentrate blood, thus achieving rapid hemostasis.

### 3.2. Water Absorption

The water absorption capacity is a key parameter for medical hemostatic materials. A high water absorption dressing can absorb exudates to concentrate blood cells and accelerate blood coagulation [20]. Table 1 shows that the water absorption rates of CS-C/β-GP and CS-C/OP/β-GP hydrogel samples were 554.77 ± 20.43% and 584.03 ± 13.16% (n = 3), respectively, indicating an excellent water absorption capacity. When the hydrogel was applied to wounds, it could absorb a large amount of water from the outflowing blood, leading to blood concentration, thereby achieving rapid hemostasis. 

### 3.3. In Vitro Hemostasis 

#### 3.3.1. Blood Coagulation Index

**Figure 2:**
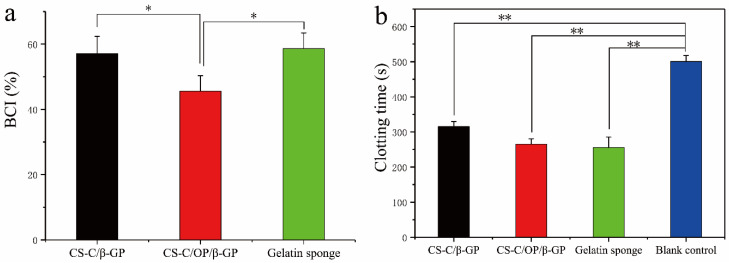
Blood coagulation index (**a**) and clotting time (**b**) in vitro, * *p* < 0.05 and ** *p* < 0.01.

The whole blood coagulation index (BCI) primarily reflects the coagulation effect of dressings. The lower the BCI is, the better the coagulation effect of the dressing [27,32,33]. The BCI of gelatin sponge was 58.25 ± 5.10% (n = 3), and the values of CS-C/β-GP and CS-C/OP/β-GP were 57.12 ± 5.20% and 45.35 ± 5.45% (n = 3), respectively. As shown in Figure 2a, the BCI of CS-C/β-GP was basically the same as that of gelatin sponge, whereas the BCI of CS-C/OP/β-GP was lower than that of gelatin sponge and CS-C/β-GP, and there were significant differences between them (*p* < 0.05), indicating that CS-C/β-GP and OP exerted a synergistic effect to promote blood clotting. The results demonstrated that CS-C/OP/β-GP achieved a good coagulation effect.

#### 3.3.2. In Vitro Procoagulant Activity

The in vitro procoagulant activity is used to evaluate the ability of a dressing to promote blood coagulation after contacting the blood. A shorter coagulation time indicates a better hemostatic effect [34,35]. The clotting time of the CS-C/β-GP, CS-C/OP/β-GP, gelatin sponge, and blank control groups was 315 ± 14 s, 265 ± 15 s, 255 ± 30 s, and 500 ± 16 s (n = 3), respectively (Figure 2b). Compared with the blank control group, both CS-C/β-GP and CS-C/OP/β-GP exhibited highly significant differences (*p* < 0.01). In particular, CS-C/OP/β-GP had a good coagulation effect equivalent to that of the gelatin sponge, and was significantly different from CS-C/β-GP (*p* < 0.05). The results suggested that CS-C/OP/β-GP could significantly shorten the coagulation time in vitro.

#### 3.3.3. Platelet Adhesion

**Figure 3:**
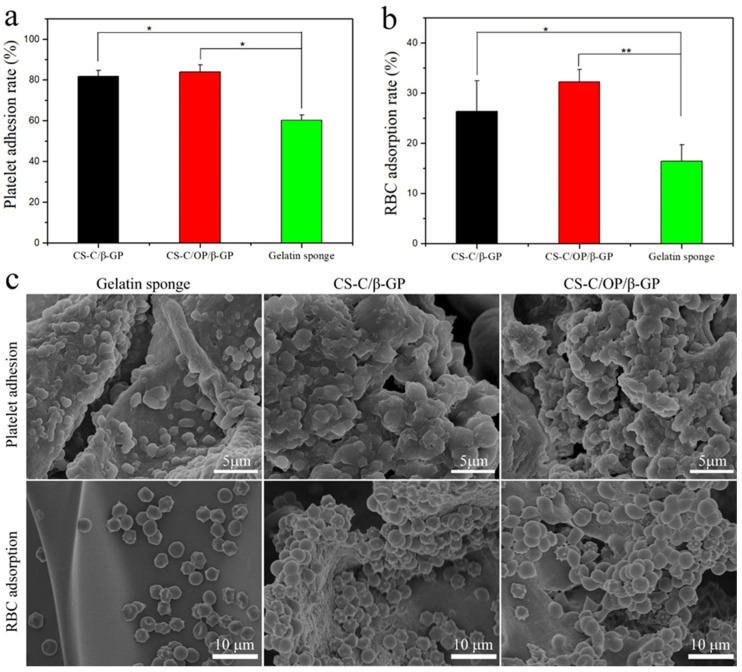
The platelet adhesion rate (**a**), red blood cell (RBC) adsorption rate (**b**), and SEM images of red blood cell adsorption and platelet adhesion (**c**), * *p* < 0.05 and ** *p* < 0.01.

Platelet adhesion of the dressing was calculated by detecting LDH activity after lysing adherent platelets [27]. As shown in Figure 3a, the platelet adsorption rates of CS-C/β-GP, CS-C/OP/β-GP, and gelatin sponge were 81.82 ± 2.84%, 83.85 ± 3.58%, and 60.33 ± 2.51% (n = 3), respectively. The platelet adhesion rate of the CS-C/OP/β-GP group was 38.98% higher than that of the gelatin sponge group (*p* < 0.05). SEM (Figure 3c) showed that more platelets and platelet aggregates were adhered to the CS-C/OP/β-GP sample with tighter adhesion compared with the gelatin sponge. This may be because the platelets that adhered to the sample were activated to facilitate the aggregation of more platelets to form clots, and finally achieved rapid hemostasis. These results indicated that CS-C/OP/β-GP could adhere to platelets, speed up platelet activation, and facilitate platelet aggregation [35].

#### 3.3.4. Erythrocyte Adsorption 

The ability of dressings to adsorb red blood cells is essential to hemostasis. This can accelerate blood clot formation at the wound, and more importantly, adherent red blood cells can deform and expose the procoagulant phospholipid (phosphatidylserine) on the membrane surface, which is similar to activated platelets [36]. As shown in Figure 3b, the erythrocyte adsorption rates of CS-C/β-GP, CS-C/OP/β-GP, and gelatin sponge were 26.38 ± 6.11%, 32.26 ± 2.45%, and 16.47 ± 3.24% (n = 3), respectively. The erythrocyte adsorption rate of CS-C/OP/β-GP was 95.87% higher than that of the gelatin sponge (*p* < 0.01), as well as significantly different from CS-C/β-GP (*p* < 0.05). This may be because the sample dressing has a good water absorption capacity and can promote the adsorption of red blood cells. The red blood cell adsorption results from SEM are shown in Figure 3c. As can be seen in Figure 3c, the surface of the gelatin sponge was smooth, so the adsorbed red blood cells were scattered and their morphology was basically normal, indicating that the erythrocyte adsorption capacity of the gelatin sponge was weak. However, more red blood cells were adsorbed on the surface of CS-C/OP/β-GP. Furthermore, it could be seen that there were small tentacles on the red blood cells at the dense places that could connect red blood cells with each other. Due to the porous structure, polycation properties, and good water absorption capacity, CS-C/OP/β-GP could absorb the water in the plasma to concentrate the blood, adsorb the red blood cells for the wound, and form a blood clot, thus achieving rapid hemostasis [37].

### 3.4. Hemostasis Activities In Vivo

#### 3.4.1. Mouse Liver Hemostasis

**Figure 4:**
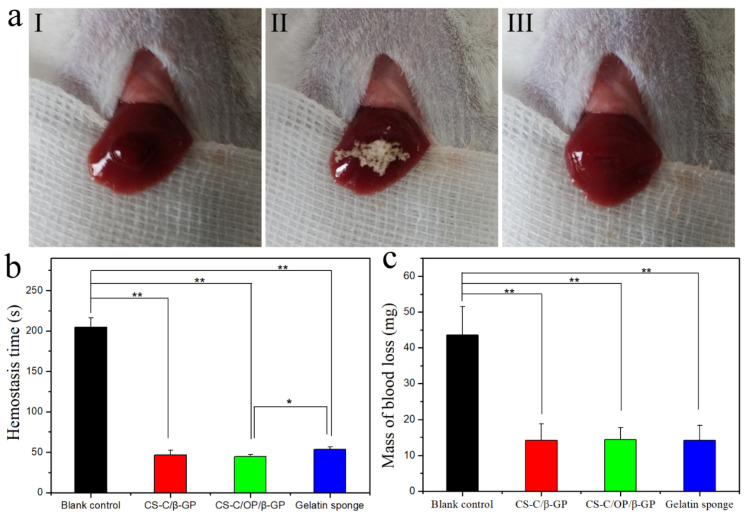
Hemostasis of the liver laceration (**a**) (I: liver bleeding; II: liver hemostasis; III: wound after hemostasis), hemostasis time (**b**), and mass of blood loss (**c**) in a liver injury model, * *p* < 0.05 and ** *p* < 0.01.

Given the widespread presence of blood vessels in various organs and the unique structure of the liver, it is difficult to ensure that all organs are sutured and deliver effectively applied pressure techniques. Therefore, liver laceration and subsequent blood loss carry a high risk of death [38]. It has been reported that chitosan and catechol-functionalized chitosan can deliver a certain hemostatic effect, by which hydrogel can adhere to the surrounding tissues and rapidly solidify to stop bleeding, thereby promoting hemostasis [6,12]. Therefore, we investigated the hemostatic effect of CS-C/OP/β-GP on a mouse liver laceration. Figure 4a shows the application of samples in the hemostasis of liver injury and the hemostasis time and liver blood losses are shown in Figure 4b,c. The blank control group without any treatment was still bleeding after 3.5 min, while hemostasis was achieved in the sample and gelatin sponge groups within 1.0 min, which indicated a highly significant difference between the sample and blank control groups (*p* < 0.01). Compared with the gelatin sponge, the hemostatic time of CS-C/OP/β-GP was 44.6 ± 2.6 s (n = 6), so was shortened by 19.5%, which was significant (*p* < 0.05). With regard to blood loss, CS-C/OP/β-GP and gelatin sponge groups displayed no significant difference, but there was a highly significant difference compared to the blank control group (*p* < 0.01). Even without applying pressure, CS-C/OP/β-GP hydrogel quickly adhered to the wound tissues to achieve rapid hemostasis. It can be concluded that CS-C/OP/β-GP hydrogel had an excellent liver hemostatic effect and greatly reduced the amount of organ bleeding. 

**Figure 5:**
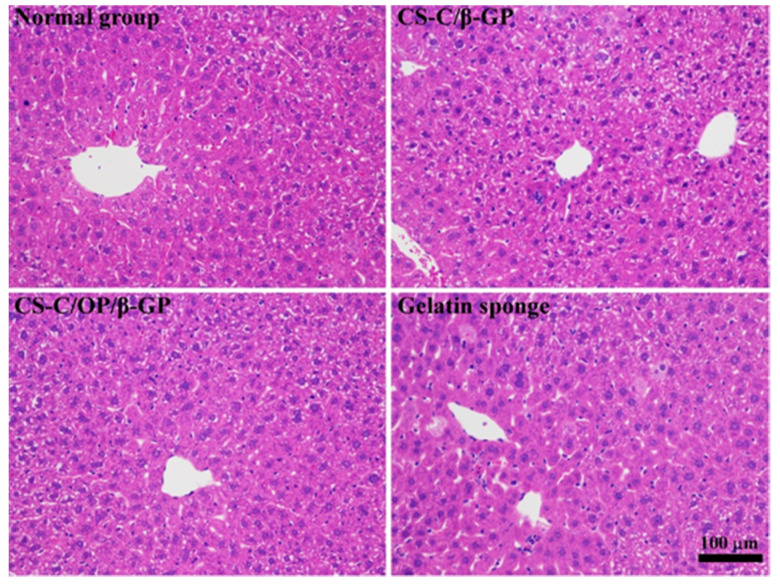
Hematoxylin and eosin (H&E) stained micrographs of liver tissues.

Figure 5 shows the liver tissue pathology after hemostasis. Normal liver parenchyma was seen in both sample groups, the liver lobule structure was well-preserved, and the central vein could be observed in the lobule. Liver cells normally radiate along the central vein, forming a plate with the thickness of a single hepatocyte. The sinusoids between the plates were normal in shape, without obvious hyperemia.

#### 3.4.2. Hemostasis of Mouse Tail Amputation

**Figure 6:**
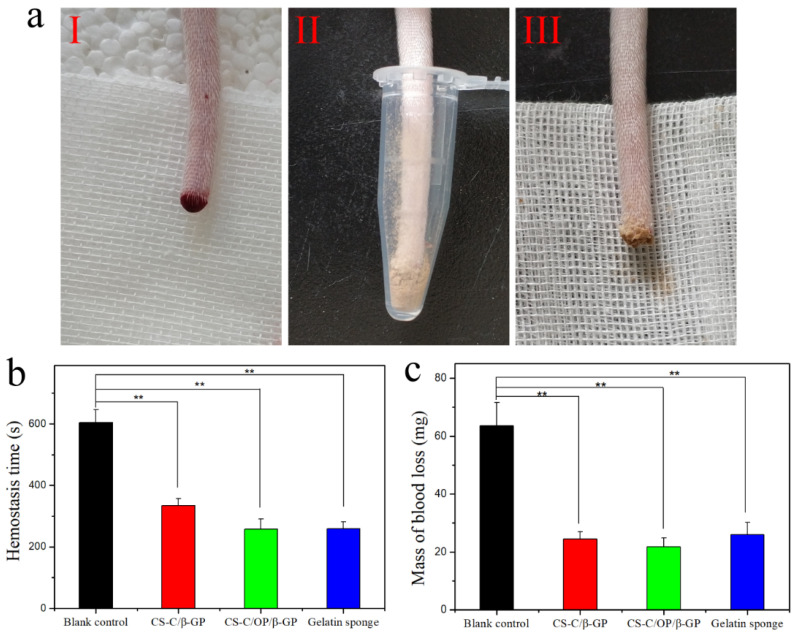
Hemostasis of the tail amputation (**a**) (I: bleeding; II: hemostasis; III: wound after hemostasis), hemostasis time (**b**), and mass of blood loss (**c**) on tail amputation, ** *p* < 0.01.

The hemostatic effect of the CS-C/OP/β-GP hydrogel was further evaluated in the mouse tail bleeding model, and the results are shown in Figure 6. Compared with the blank control group, the hemostasis time and mass of blood loss of the gelatin sponge, CS-C/β-GP, and CS-C/OP/β-GP groups were significantly reduced (*p* < 0.01). Furthermore, the hemostasis time of CS-C/OP/β-GP hydrogel was not significantly different from the gelatin sponge group, but the mass of blood loss decreased by 18.9%.

### 3.5. Safety Evaluation

#### 3.5.1. Cytotoxicity

**Figure 7:**
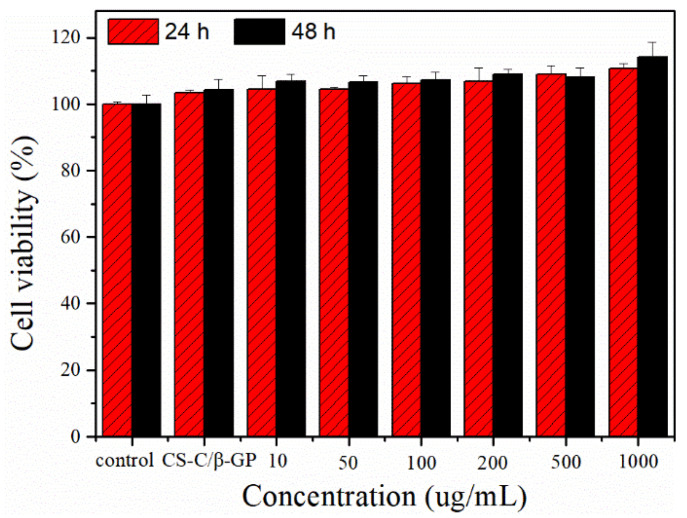
Effects of CS-C/OP/β-GP on the L929 cell viability.

In the early stages of wound healing, fibroblasts play a vital role by actively proliferating, migrating to the wound area, and inducing the synthesis of new extracellular matrix and thick actin myofibroblasts [39]. The L929 fibroblast cell line has been extensively employed as an in vitro model of skin cell behavior [15,40]. L929 cells treated by CS-C/OP/β-GP hydrogel with different concentrations were cultured for 24 and 48 h, and the cell viability results are shown in Figure 7. Compared with the control group, CS-C/OP/β-GP with different concentrations had a higher cell viability, indicating that CS-C/OP/β-GP was non-toxic to L929 cells. Compared with CS-C/β-GP, the cell viability of CS-C/OP/β-GP at different concentrations was higher, and it exhibited a dose-dependent and time-dependent manner, which could be reasonably explained by the fact that OP delivered some nutrients to promote cell proliferation.

**Figure 8:**
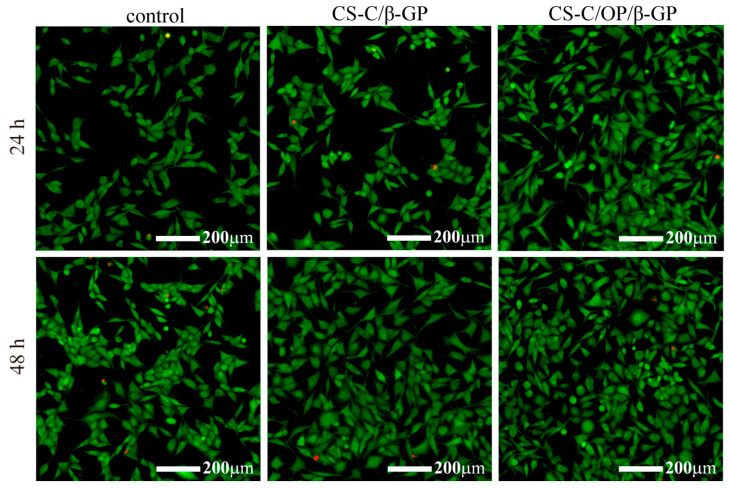
Calcein-AM/PI double staining for L929 cells.

The cytotoxicity of the samples in L929 cells was further evaluated by the double staining of Calcein-AM/PI. The green fluorescent signal represents living cells, and the red signal indicates dead cells [41]. As shown in Figure 8, there was no significant difference between the control group and the samples at 24 h, whereas CS-C/β-GP and CS-C/OP/β-GP displayed strong green fluorescence compared with the control group at 48 h. From the pictures of the samples, except for the individual dead cells, most of the cells maintained a normal spindle shape and had a higher cell density and even distribution. These results indicated that L929 cells could grow normally in the presence of CS-C/OP/β-GP.

**Figure 9:**
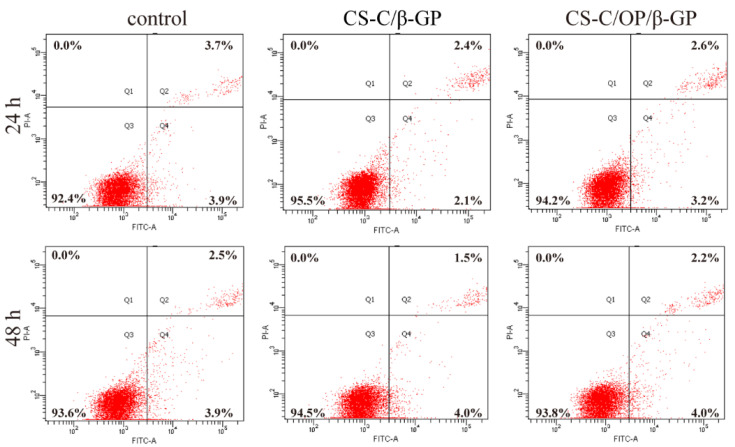
Analysis of L929 cell apoptosis by flow cytometry.

L929 cell apoptosis was quantitatively analyzed by flow cytometry with the Annexin FITC/PI double staining method. As shown in Figure 9, the apoptosis rates of the control group were 7.6% and 6.4% at 24 and 48 h, respectively. After 24 h treatment, the apoptosis rates of CS-C/β-GP and CS-C/OP/β-GP were 4.5% and 5.8%, respectively. After 48 h, those were 5.5% and 6.2%, respectively. Compared with the control group, the apoptosis rates of CS-C/β-GP and CS-C/OP/β-GP hydrogel groups were lower, but there were no significant differences among them. The results indicated that CS-C/OP/β-GP had no significant effects on L929 cell apoptosis.

#### 3.5.2. Hemolysis Rate

**Figure 10:**
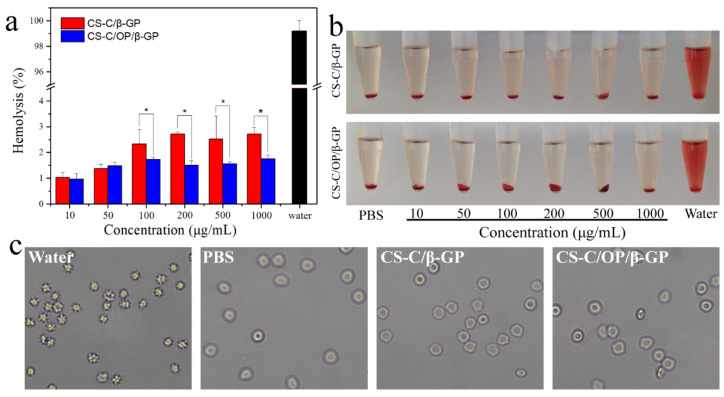
(**a**) Hemolysis ratio, (**b**) RBC suspension solutions, and (**c**) microscopy of RBCs treated with samples, * *p* < 0.05.

A low hemolysis rate of a material suggests that it causes limited damage to red blood cells and has better blood compatibility [42]. As shown in Figure 10a, the hemolysis rates of hydrogel samples increased slightly with increasing concentrations. However, within 1000 μg/mL, the hemolysis rates were all less than 5%, indicating good blood compatibility. From Figure 10b, the supernatants of red blood cell suspensions were clear or light pink, indicating no or slight hemolysis. The morphological changes of treated red blood cells are shown in Figure 10c. It can be seen that the cells in the negative control group (PBS) were intact and disc-shaped, while those in the positive control group (water) were broken. After treatment with different concentrations of hydrogels, the morphology of red blood cells was similar to that of the PBS group, showing that there was no obvious hemolytic effect. This demonstrated that hydrogel samples within 1000 μg/mL did not cause hemolysis and had good blood compatibility.

## 4. Conclusions

With its porous three-dimensional network structure, CS-C/OP/β-GP thermo-sensitive hydrogel can quickly absorb water and concentrate blood. In vitro testing revealed that CS-C/OP/β-GP hydrogel adsorbed a large number of red blood cells to form blood clots and promoted blood clotting. Meanwhile, CS/OP/β-GP hydrogel adhered to platelets, accelerated platelet activation, facilitated platelet aggregation, and led to rapid hemostasis. In mouse hemorrhagic models, CS-C/OP/β-GP hydrogel exerted a sealing effect on the wound surface with a short hemostasis time and less blood loss than the medical gelatin sponge, thus achieving rapid hemostasis. The rapid and effective hemostasis of CS-C/OP/β-GP hydrogel may be attributed to the three-dimensional porous structure of the material, the multiple coagulation pathways of chitosan independent of the classical coagulation cascade, and the activation of endogenous coagulation factors by oyster peptides. As for the specific hemostasis mechanisms, this still remains a great challenge. Safety evaluations confirmed that CS-C/OP/β-GP hydrogel was not cytotoxic and had desirable blood compatibility. In conclusion, CS-C/OP/β-GP hydrogel is expected to be developed as a hemostatic dressing.

## Figures and Tables

**Table 1:** Water absorption rate of freeze-dried hydrogels.

Sample	Water Absorption Rate (%)
CS-C/β-GP	554.77 ± 20.43
CS-C/OP/β-GP	584.03 ± 13.16
